# Consumption of Non-Nutritive Sweetener, Acesulfame Potassium Exacerbates Atherosclerosis through Dysregulation of Lipid Metabolism in ApoE^−/−^ Mice

**DOI:** 10.3390/nu13113984

**Published:** 2021-11-09

**Authors:** Cheng-Hsin Lin, Hung-Yuan Li, Shu-Huei Wang, Yue-Hwa Chen, Yang-Ching Chen, Hung-Tsung Wu

**Affiliations:** 1 Department of Surgery, Shuang Ho Hospital, College of Medicine, Taipei Medical University, Taipei 110, Taiwan; chlin99025@tmu.edu.tw; 2 Department of Internal Medicine, National Taiwan University Hospital, Taipei 100, Taiwan; larsli@ntuh.gov.tw; 3 Department of Anatomy and Cell Biology, College of Medicine, National Taiwan University, Taipei 100, Taiwan; shwang@ntu.edu.tw; 4 School of Nutrition and Health Sciences, Taipei Medical University, Taipei 110, Taiwan; yuehwa@tmu.edu.tw; 5 School of Food Safety, Taipei Medical University, Taipei 110, Taiwan; 6 Department of Family Medicine, Taipei Medical University Hospital, Taipei 110, Taiwan; melisa26@tmu.edu.tw; 7 Department of Family Medicine, School of Medicine, College of Medicine, Taipei Medical University, Taipei 110, Taiwan; 8 Department of Internal Medicine, School of Medicine, College of Medicine, National Cheng Kung University, Tainan 701, Taiwan

**Keywords:** acesulfame potassium, apolipoprotein E, atherosclerosis, dysregulation, lipid metabolism

## Abstract

Obesity is associated with the risk of cardiovascular disease, and non-nutritive sweetener, such as acesulfame potassium (AceK) has been used to combat obesity. However, the effects of AceK on cardiovascular disease are still unclear. In this study, high cholesterol diet (HCD)-fed ApoE^−/−^ mice had dysregulated plasma lipid profile, and developed atherosclerosis, determined by atherosclerotic plaque in the aorta. Supplement of AceK in HCD worsened the dyslipidemia and increased atherosclerotic plaque, as compared with HCD-fed ApoE^−/−^ mice. Since treatment of AceK in RAW264.7 macrophages showed no significant effects on inflammatory cytokine expressions, we then investigated the impacts of AceK on lipid metabolism. We found that AceK consumption enhanced hepatic lipogenesis and decreased β-oxidation in ApoE^−/−^ mice. In addition, AceK directly increased lipogenesis and decreased β-oxidation in HepG2 cells. Taken together, a concurrent consumption of AceK exacerbated HCD-induced dyslipidemia and atherosclerotic lesion in ApoE^−/−^ mice, and AceK might increase the risk of atherosclerosis under HCD.

## 1. Introduction

Obesity is a multifactorial disease that is linked with high prevalence rates of non-communicable diseases [1,2,3]. The growing trend of obesity makes it as a serious public health threat that exhibits no boundaries [4]. The global prevalence of obesity has almost tripled since 1975 and obesity has a serious impact on human health [4]. Ample evidence has disclosed that sugar consumption is fueling the epidemic of obesity, elevating the risk of obesity-related morbidities [5]. Additionally, obesity contributes directly to the incidence of cardiovascular risk factors, including dyslipidemia, type 2 diabetes and hypertension [6]. Yet, cardiovascular disease is one of the leading causes of death globally [7]. Therefore, the public health issue of obesity should be taken seriously.

Recently, non-nutritive sweetener (NNS) has been widely used as a substitute for table sugar [8]. NNS is non-nutritive and contains no calories, attributes that could make it useful against obesity pandemic [9]. NNS is hundreds or thousands of times sweeter than table sugar and can provide a lingual sweet taste sensation without an excessive calorie load. These benefits have accelerated the use of NNS over the past decades [10,11]. In parallel to the dramatic increase in the consumption of NNS sweetened food, concerns have been raised about their potential adverse health effects. Several studies have proposed that NNS consumption may contribute to a profound impact on glucose homeostasis and insulin resistance, promoting excessive energy consumption from NNS-sweetened food products that even produce diet-induced obesity [11,12]. Other health hazards, such as brain tumor, cancer and dysbiosis of gut microbiota have also been proposed [12]. Thus, the health concerns are greater than the prospective beneficial effects of chronic NNS consumption.

Acesulfame potassium (AceK) is a chemically produced sweetener which belongs to one of five Food and Drug Administration-approved NNS that could be used as an additive in foods and beverages [13]. AceK is a heat-stable compound and highly soluble in water. It is 200 times sweeter than table sugar which is commonly used in baked goods, frozen desserts, candy, etc. [8,12]. Recently, some findings have suggested that chronic consumption of AceK plays a crucial role in intestinal glucose uptake and adipogenesis in murine models [11,14]. Cognitive impairment and genotoxicity effects induced by AceK consumption further challenge the safety use of AceK as a food additive [15,16,17]. Long-term consumption of AceK may induce hyperlipidemia in wild type mice [1]. However, the mechanism underlying the effects of AceK on atherosclerosis remains unknown. Thus, in the present study, we aimed to investigate the effects of AceK on the development of atherosclerosis in apolipoprotein E deficient (ApoE^−/−^) mice.

## 2. Material and Methods

### 2.1. Experimental Animals

Eight-week-old male ApoE^−/−^ mice were purchased from National Laboratory Animal Center (Taipei, Taiwan) and housed in Laboratory Animal Center, Taipei Medical University, Taiwan under barrier-maintained conditions (temperature: 22 ± 2 °C, humidity: 55%, 12:12 h light:dark cycle). The animals were randomly assigned to four groups (*n* = 9 per group): Group I (Chow), ApoE^−/−^ mice fed with standard chow diet (2.89 kcal/g) (Laboratory Rodent Diet 5001, LabDiet, St. Louis, MO, USA); Group II (AceK), ApoE^−/−^ mice fed with chow diet and AceK; Group III (high cholesterol diet, HCD), ApoE^−/−^ mice fed with HCD; Group IV (HCD-AceK), ApoE^−/−^ mice fed with HCD and AceK. The HCD contained 21% fat and 0.15% cholesterol (4.67 kcal/g) (D12079B, Research diet, New Brunswick, NJ, USA) and was given for 8 weeks to accelerate the development of atherosclerosis in ApoE^−/−^ mice. AceK (Sigma-Aldrich, St. Louis, MO, USA) of 15 mg/kg body weight per day, which is equal to the acceptable daily intake (ADI), was dissolved in sterile saline and administrated by oral gavage once daily [18]. The food intake was measured weekly and converted into caloric intake.

### 2.2. Morphological Analysis

The heart and aortic tissue were harvested and fixed with 10% formalin according to a previous study [19]. The tissue was embedded in optimal cutting temperature compound and cut into 3 µm sections for histological measurement of atherosclerotic lesions in aortic sinus. Oil red O staining (Sigma-Aldrich, St. Louis, MO, USA) was used to visualize the atherosclerotic lesion area. Lesion area was quantified using imageJ software on 12 July 2020 (https://imagej.nih.gov/ij/).

### 2.3. Biochemical Analysis

The mice were fasted for 12 h and then blood samples were collected for the determination of lipid profile using commercialized assay kits. Serum triglyceride concentrations were measured using triglyceride colorimetric assay kit (Cayman, Ann Arbor, MI, USA) and total cholesterol, low-density lipoprotein cholesterol (LDL-cholesterol) and high-density lipoprotein cholesterol (HDL-cholesterol) were measured using EnzyChrom^TM^ AF HDL and LDL/VLDL assay kits (Bioassay System, Hayward, CA, USA).

### 2.4. Cell Culture

*Mus musculus* macrophage cell line (RAW264.7) and *Homo Sapiens* hepatocellular carcinoma cell line (HepG2) were purchased from the Bioresource Collection and Research Center (Hsinchu, Taiwan). RAW264.7 murine macrophage cells were grown in 25 cm^2^ flasks in RPMI-1640 medium containing 10% fetal bovine serum with penicillin (100 U/mL) and streptomycin (100 μg/mL). HepG2 cells were maintained in Dulbecco’s Modified Eagle Medium (Gibco, Amarillo, TX, USA) with 10% fetal bovine serum and 100 units/mL penicillin, and 100 μg/mL streptomycin.

### 2.5. Real-Time Polymerase Chain Reactions

The cells were subjected to ribonucleic acid (RNA) isolation using GENEzol™ TriRNA Pure Kit (Geneaid Biotech, New Taipei City, Taiwan). Two μg of RNA were used to react with Moloney murine leukemia virus reverse transcriptase (ProTech, Taipei, Taiwan) to generate complementary deoxyribonucleic acid. Polymerase chain reaction (PCR) amplification was performed using the target primers and Luna Universal qPCR Master Mix (New England Biolabs, Ipswich, MA, USA) for 10 min at 95 °C, followed by 40 cycles set for 10 s at 95 °C, annealing for 10 s at 65 °C, and extending for 2 s at 72 °C. Glyceraldehyde 3-phosphate dehydrogenase (GAPDH) was used as an internal control. The sequences of the target primers were shown as following. *Mus musculus* tumor necrosis factor-α (*Tnf*): forward: 5′- GGT GCC TAT GTC TCA GCC TCT T -3′, reverse: 5′- GCC ATA GAA CTG ATG AGA GGG AG-3′; *Mus musculus* interleukin 6 (*Il-6*): forward: 5′-TAC CAC TTC ACA AGTCG GAG GC-3′, reverse: 5′- CTG CAA GTG CAT CAT CGT TGT TC-3′; *Mus musculus* C-C Motif Chemokine Ligand 2 (*Ccl2*): forward: 5′- GCT ACA AGA GGA TCA CCA GCA G-3′, reverse 5′-GTC TGG ACC CAT TCC TTC TTG G-3′; *Mus musculus Gapdh*: 5′- CAT CAC TGC CAC CCA GAA GAC TG-3′, reverse: 5′- ATG CCA GTG AGC TTC CCG TTC AG-3′. *Homo sapiens* acetyl-coA carboxylase (*ACC*): forward: 5′-ACA TTC CCT GAG GCA GGT CA-3′, reverse: 5′-GAT CCC ATG GTC AAC CAG GG-3′; *Homo sapiens* fatty acid synthase (*FASN*): forward: 5′-CGG TGT GTG CTG CTC TCC AA-3′, reverse: 5′-CAG CAG GAA GTG GCG GAA AG-3′; *Homo sapiens* sterol regulatory element binding protein-1 (*SREBP1*): forward: 5′ -GGG CCT TGC ATT TTC TGA CA-3′, reverse: 5′-CAC GAA GAA ACG GTG GCC CA-3′; *Homo sapiens* peroxisomal acyl-coenzyme A oxidase (ACOX): forward: 5′-GGC GCA TAC ATG AAG GAG ACC T-3′, reverse: 5′-AGG TGA AAG CCT TCA GTC CAG C-3′; *Homo sapiens* carnitine palmitoyltransferase 2 (*CPT2*): forward: 5′-CCCTGCATACCAGCGGATAA-3′, reverse: 5′-CATACGCAATGCCAAAGCCA-3′; *Homo sapiens* peroxisome proliferator-activated receptor-α (*PPARA*): forward: 5′-GCC TGT CTG TCG GGA TGT-3′, reverse: 5′-GGC TTC GTG GAT TCT CTT G-3′; 3-hydroxy-3-methyl-glutaryl-coenzyme A reductase (*HMGCR*): forward: 5′-GAC GTG AAC CTA TGC TGG TCA G-3′, reverse: 5′-GGT ATC TGT TTC AGC CAC TAA GG-3′; *Homo sapiens* GAPDH: 5′-TGG AAA TCC CAT CAC CAT CT-3′, reverse: 5′-GTC TTC TGG GTG GCA GTG AT-3′.

### 2.6. Western Blot Analysis

The samples were lysed using radioimmunoprecipitation assay buffer containing protease inhibitors (Sigma-Aldrich, St. Louis, MO, USA). Protein concentrations were determined using bicinchoninic acid protein assay (G-Biosciences, Maryland Heights, MO, USA). Thirty µg of protein lysates were separated using sodium dodecyl sulfate-polyacrylamide gel electrophoresis and transfer onto polyvinylidene fluoride membrane (Millipore, Burlington, MA, USA). The membranes were blocked at room temperature for one hour using TBS-T (10 mM Tris, 150 mM NaCl, and 0.05% Tween 20, (pH 7.6)) containing 10% skimmed milk, then probed with 1:1000 primary antibodies, such as FAS (Abcam, Cambridge, UK), ACC (Cell signaling, Danvers, MA, USA), SREBP1 (Novus, Centennial, CO, USA), ACOX1, CPT2 (Affinity Biosciences, Pottstown, PA, USA), PPARα (GeneTex, Alton Pkwy Irvine, CA, USA), HMGCR (Abcam, Cambridge, UK) and actin (Abnova, Taipei, Taiwan) at 4 °C overnight. Blots were then washed with TBS-T and incubated with a 1:5000 dilution of horseradish peroxidase-conjugated secondary antibodies (Abcam, Cambridge, UK) at room temperature for one hour. Protein bands were visualized using a chemiluminescence horseradish peroxidase substrate (Millipore, Burlington, MA, USA), and the relative signal intensity was quantified using ImageJ software.

### 2.7. Statistical Analysis

GraphPad Prism 6 was used for all statistical analyses. Data was analyzed using unpaired Student’s *t-*test and presented as the mean ± standard error of the mean (SEM), and significance levels were indicated at *p* < 0.05.

## 3. Results

### 3.1. AceK Exacerbated Atherosclerosis in High Cholesterol Diet Fed ApoE^−/−^ Mice

**Figure 1:**
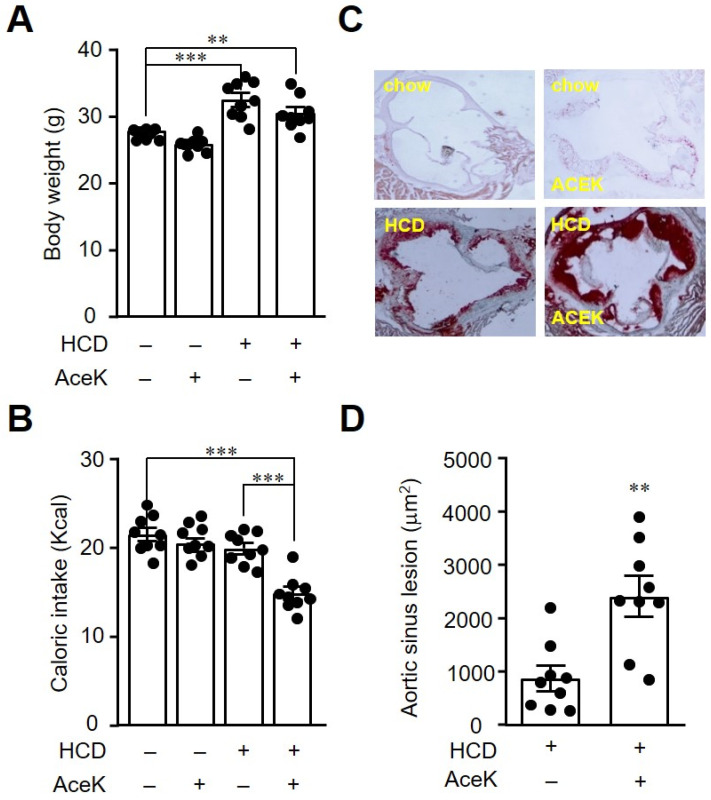
AceK exacerbated atherosclerosis in high cholesterol diet-fed ApoE^−/−^ mice. Mice were fed with chow diet or high cholesterol diet (HCD) for 8 weeks with or without 15 mg/kg AceK administration once daily. The body weight (**A**), and calorie intake (**B**) were recorded. The aortic sinus sections were stained with Oil Red O to visualize the atherosclerotic formed (**C**), and the quantification of the aortic sinus lesion area by imageJ (**D**). ** *p* < 0.01, *** *p* < 0.001.

After an eight-week feeding of HCD with or without AceK, body weight showed a significantly increase in HCD group, as compared with Chow group, whereas there were no significant differences between HCD group, and HCD-AceK group (Figure 1A). In addition, we found a significant decrease of daily calorie intake in HCD-AceK group (Figure 1B). To determine the effects of AceK on the development of atherosclerosis, we then measured the atherosclerotic plaque formed in aortic sinus. It was known that HCD accelerated the development of atherosclerosis, as compared with chow diet in ApoE^−/−^ mice. In this study, we found mild atherosclerotic plaque in chow-fed ApoE^−/−^ mice at the age of sixteen-weeks-old. However, a notably atherosclerotic plaque was formed in the aortic sinus in HCD-fed ApoE^−/−^ mice. AceK intervention further exacerbated the development of atherosclerosis (Figure 1C,D). We therefore examined the aortic sinus lesion area in both groups of HCD-fed ApoE^−/−^ mice and HCD-fed AceK supplemented ApoE^−/−^ mice (Figure 1D). The aortic sinus lesion area was significantly increased in HCD-fed AceK supplemented ApoE^−/−^ mice, as compared with HCD-fed mice, indicating AceK may accelerate the development of atherosclerosis.

### 3.2. AceK Showed No Significant Effects on Proinflammatory Cytokine Expressions in RAW264.7 Macrophages

**Figure 2:**
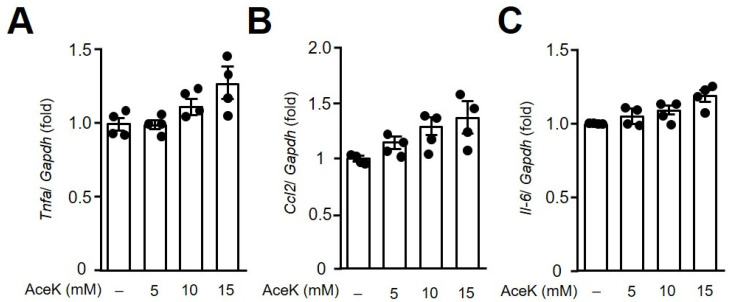
Effects of AceK on proinflammatory cytokine expressions in murine RAW264.7 macrophages. Cells were treated with indicated doses of AceK for 24 h. The cells were harvested and RNA was isolated for the quantification of tumor necrosis factor alpha (*Tnfa*) (**A**), C–C motif chemokine ligand 2 (*Ccl2*) (**B**), and interleukin-6 (*Il-6*) (**C**) gene expressions by quantitative PCR (*n* = 4).

The underlying pathogenesis of atherosclerosis encompassed an imbalanced lipid metabolism and a maladaptive immune response entailing a chronic inflammatory response in the arterial wall. The persistent inflammatory signals further lead to an endothelial dysfunction. We therefore investigated the inflammatory cytokine expressions in responses to AceK treatment in murine macrophages. As shown in Figure 2, treatment of AceK at various doses in RAW264.7 for 24 h, the expressions of *Tnfa* (Figure 2A), *Ccl2* (Figure 2B) and *Il-6* (Figure 2C) showed no significant differences between AceK treated and untreated groups, implying inflammation might not be involved in AceK-accelerated atherosclerosis.

### 3.3. AceK Worsened Dyslipidemia in High Cholesterol Diet-Fed ApoE^−/−^ Mice

**Figure 3:**
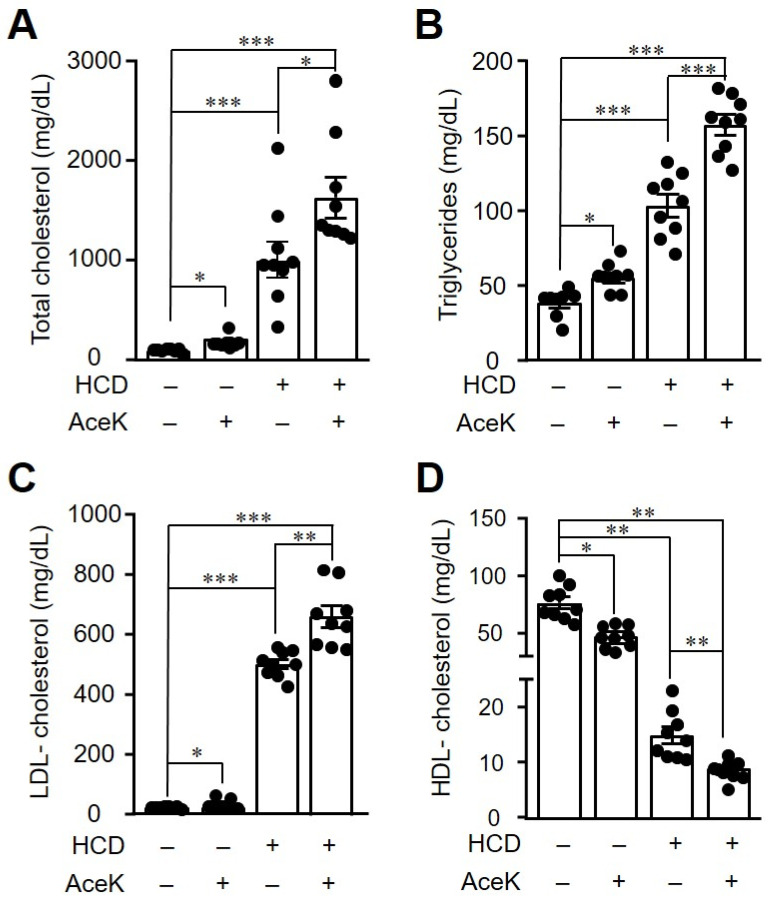
Administration of AceK worsened dyslipidemia in high cholesterol diet-fed ApoE^−^^/^^−^ mice. The mice were fasted for 12 h and then blood samples were collected for the determination of lipid profiles. Plasma total cholesterol (**A**), triglyceride (**B**), low-density lipoprotein cholesterol (LDL-cholesterol) (**C**), and high-density lipoprotein cholesterol (HDL-cholesterol) (**D**) were determined using commercialized assay kits (*n* = 9 per group of mice). * *p* < 0.05, ** *p* < 0.01, *** *p* < 0.001.

Accumulating studies disclosed that dyslipidemia can provoke endothelial dysfunction by aggregating immune cells on the arterial wall [20]. In order to investigate the effects of AceK on lipid homeostasis, we then analyzed the lipid profile in ApoE^−/−^ mice. Consistent with previous study, we found that AceK treatment slightly, but significantly, induced hyperlipidemia [1]. In addition, the plasma total cholesterol (Figure 3A), triglycerides (Figure 3B) and LDL-cholesterol (Figure 3C) were significantly increased in ApoE^−/−^ mice, whereas HDL-cholesterol levels were decreased (Figure 3D), as compared with Chow group. AceK intervention further increased the total cholesterol, triglyceride and LDL-cholesterol concentrations than HCD group, whereas the levels of HDL-c were further decreased (Figure 3). Together with the morphological analysis, we suggested that AceK exacerbated the development of atherosclerosis through the dysregulation of lipid metabolism.

### 3.4. AceK Impaired Lipid Homeostasis in ApoE^−/−^ Mice

**Figure 4:**
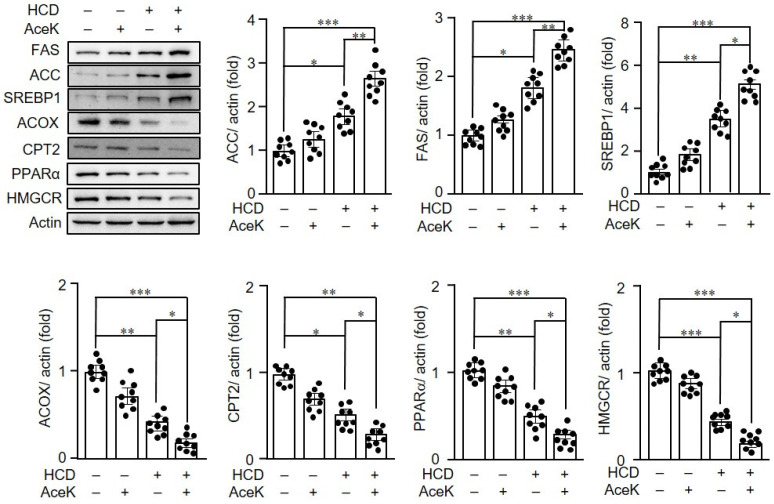
AceK dysregulated lipid homeostasis in ApoE^−/−^ mice. The protein expressions in the liver of ApoE^−/−^ mice of acetyl-coA carboxylase (ACC), fatty acid synthase (FAS), sterol regulatory element binding protein-1 (SREBP1), peroxisomal acyl-coenzyme A oxidase (ACOX), carnitine palmitoyltransferase-2 (CPT2), peroxisome proliferator-activated receptor α (PPARα) and 3-hydroxy-3-methyl-glutaryl-coenzyme A reductase (HMGCR) were determined by Western blots (*n* = 9 per group of mice). * *p* < 0.05, ** *p* < 0.01, *** *p* < 0.001.

To clarify the possible mechanisms related to lipid metabolic dysregulation induced by AceK, we investigated the key proteins in lipogenesis and lipolysis pathways. As shown in Figure 4, the lipogenesis-related proteins, such as ACC, FAS and SREBP1 protein expressions were significantly increased in HCD group as compared with Chow group. AceK intervention further significantly increased the hepatic expressions of ACC, FAS and SREBP1 than HCD group. In addition, we found that the expression of the rate-limiting enzyme for cholesterol synthesis, HMGCR, was significant decreased in HCD-fed ApoE^−/−^ mice, and AceK supplement further enhanced the effects of HCD on HMGCR expression

Lipid homeostasis is a subtle balance between lipogenesis and lipolysis [21]. Thus, we further measured the hallmark proteins in lipolysis. HCD group showed significantly decreased ACOX, CPT-2, and PPARα expressions than Chow group. The AceK intervention further augmented the effects of HCD on the hepatic protein expressions of ACOX, CPT2 and PPARα.

### 3.5. AceK-Induced Dysregulation of Lipid Homeostasis in HepG2 Cells

**Figure 5:**
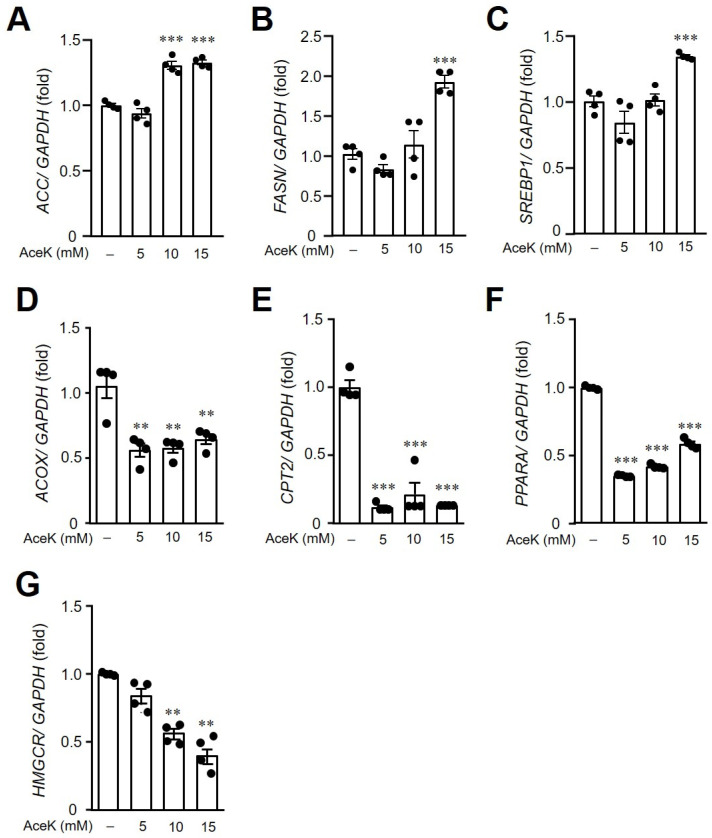
Effects of AceK on lipid metabolism-related gene expressions in HepG2 cells. HepG2 were treated with indicated doses of AceK for 24 h. The cells were then harvested and RNA was isolated for the quantification of acetyl-coA carboxylase (*ACC*) (**A**), fatty acid synthase (*FASN*) (**B**), sterol regulatory element binding protein-1 (*SREBP1*) (**C**), peroxisomal acyl-coenzyme A oxidase (*ACOX*) (**D**), carnitine palmitoyltransferase-2 (*CPT2*) (**E**), peroxisome proliferator-activated receptor-α (*PPARA*) (**F**), and 3-hydroxy-3-methyl-glutaryl-coenzyme A reductase (*HMGCR*) (**G**) gene expressions by quantitative PCR (*n* = 4). ** *p* < 0.01, *** *p* < 0.001.

**Figure 6:**
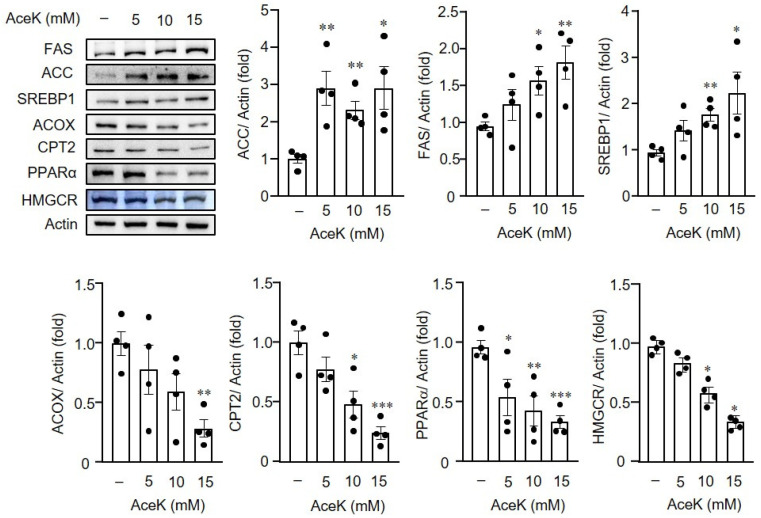
Effects of AceK on lipid metabolism-related protein expressions in HepG2 cells. HepG2 were treated with indicated doses of AceK for 24 h. The protein lysates were prepared for the quantification of acetyl-coA carboxylase (ACC), fatty acid synthase (FAS), sterol regulatory element binding protein-1 (SREBP1), peroxisomal acyl-coenzyme A oxidase (ACOX), carnitine palmitoyltransferase-2 (CPT2), peroxisome proliferator-activated receptor α (PPARα) and 3-hydroxy-3-methyl-glutaryl-coenzyme A reductase (HMGCR) protein expressions by Western blots (*n* = 4). * *p* < 0.05, ** *p* < 0.01, *** *p* < 0.001.

Following the investigation of the effects of AceK on lipid metabolism in animals, we then elucidated whether AceK directly induced the imbalance of lipid homeostasis. HepG2 cells were used and treated with different doses of AceK. AceK treated-HepG2 cells showed significant upregulation of the lipogenesis-related gene expressions, including *ACC* (Figure 5A), *FASN* (Figure 5B) and *SREBP1* (Figure 5C). On the other hand, AceK treatment in HepG2 cells showed significant lower lipolysis-related gene expressions, including *ACOX* (Figure 5D), *CPT2* (Figure 5E) and *PPARα* (Figure 5F) than control group. To further confirm the effects of AceK on lipid metabolism, we analyzed lipogenic and lipolytic protein expressions in AceK-treated HepG2 cells. In accordance with the results of lipogenic and lipolytic gene expressions, we found that AceK treatment dose-dependently increased lipogenesis-related protein expressions, and decreased lipolysis-related protein expressions (Figure 6).

## 4. Discussion

To the best of our knowledge, the present study is the first report to investigate the effects of non-nutritive sweetener, AceK on the mechanism underlying the pathogenesis of atherosclerosis. In this study, we found that AceK consumption might exacerbate HCD-induced atherosclerosis. AceK significantly increased the blood lipid levels in ApoE^−/−^ mice, which led to a severe hyperlipidemia. Moreover, an upregulation of lipogenesis-related genes alongside with a downregulation of β-oxidation-related genes resulted in an imbalance of lipid homeostasis. These effects of AceK on lipid metabolism might further augment the severity of atherosclerosis.

In accordance to the previous study, a notably atherosclerotic plaque was formed in the aortic sinus and aorta in HCD-fed ApoE^−/−^ mice [22]. In the present study, we disclosed an additional effect of AceK on the progression of atherosclerosis. We found that there were no significant differences of body weight between HCD group, and HCD AceK group, consistent with a previous study indicating that chronic ingestion of AceK has limited influence on body weight and metabolic homeostasis in C57BL/6 mice [16]. However, the calorie intake of HCD-AceK group was significantly lower than that of HCD group in ApoE^−/−^ mice without significant body weight change, implying AceK might have effects on lipid metabolism.

Atherosclerosis is a chronic inflammation and immune disease, characterized by a dysfunctional interplay between the impaired immunity and dyslipidemia [23]. Inflammation-induced endothelial dysfunction is the early key marker for atherosclerosis. We thereby treated RAW264.7 macrophages with AceK to integrate the effects of AceK on inflammation. In the present study, our results demonstrated that AceK had no significant effects on inflammatory responses in macrophages. In view of a significant higher atherosclerotic lesion area in aortic sinus after AceK consumption, we then further investigated the effects of AceK on lipid homeostasis.

Sweet taste receptors are not only the receptors sensing sweetness, but play important roles in the regulation of lipid metabolism. It was shown that both T1R2 and T1R3 knockout mice have reduced adiposity and smaller adipocytes [24], implying activation of the sweet taste receptors might facilitate lipogenesis. AceK is one of the ligands that binds to sweet taste receptors [25], and extracellular signal–regulated kinase mitogen-activated protein kinase (ERK1/2)-pathway is one of main downstream signals for sweet taste receptors [26]. Previous studies found that the activation of ERK1/2 pathway decreased cardiac PPARα gene expression and activity [27]. Although multiple mechanisms might be involved in artificial sweetener-stimulated adipogenesis and -suppressed lipolysis [28], we speculated that ACEK might regulate the levels of PPARα through activation of the sweet taste receptors, and further studies are needed to investigate the detail mechanisms related to ACEK-induced hyperlipidemia.

Ample studies have demonstrated that abnormal lipid homeostasis may overtly elevate atherosclerotic risks [29]. In the present study, a dramatic increase in plasma total cholesterol, triglyceride and LDL-cholesterol concentrations were found in ApoE^−/−^ mice fed with HCD, which conformed with the findings of previous studies [30]. Deletion of the ApoE gene caused an inability in clearance of circulatory lipid and even a boost in the sensitivity to a dietary cholesterol, making the mice suffer a severe hypercholesterolemia [30]. In addition, we found that AceK might regulate the lipid metabolism, including lipogenesis and lipolysis to exacerbate hyperlipidemia in HCD AceK group. On the other hand, a study indicated that NNS consumption may be attributable to endocrine metabolism [13]. Inhibition of cholesterol clearance by ApoE knockout showed a feedback effect to decrease HMGCR expressions in ApoE^−/−^ mice [31,32]. In the present study, we found that AceK treatment decreased the expressions of HMGCR, consistent with a previous study indicating that AceK might alter the structure of HDL-cholesterol, and decrease the binding ability of the lipoprotein, thereby affecting the lipid clearance activity in the body [33]. Thus, not only the effects of Acek on lipogenesis and lipolysis might be related to the AceK-exacerbated atherosclerotic, but also the cholesterol clearance activity.

Although ApoE^−/−^ mice are extensively used for atherosclerosis research, there are still several limitations [34,35], and the model does not compare well to human in terms of lipid profile. Thus, the effects of AceK on the development of atherosclerosis in human still need further studies to investigate.

## 5. Conclusions

Taken together, we found that AceK augmented HCD-induced dyslipidemia through an increment in lipogenesis and decrement in lipolysis, and these effects might further exacerbate HCD-induced atherosclerosis in ApoE^−/−^ mice. Thus, AceK posed additional effects on lipogenesis alongside with HCD. AceK might disturb lipid metabolism in the liver which further contributes to lipid dysregulation thereby enhancing the progression of atherosclerosis.

## Data Availability

Data is contained within the article.

## Abbreviations

AceK	Acesulfame potassium
ACC	Acetyl-coA carboxylase
ApoE^−/−^	Apolipoprotein E deficient
CPT2	Carnitine palmitoyltransferase-2
FAS	Fatty acid synthase
GAPDH	Glyceraldehyde 3-phosphate dehydrogenase
HCD	High cholesterol diet
HUVECs	Human umbilical vein endothelial cell line
HDL-c	High-density lipoprotein cholesterol
IL-6	Interleukin 6
LDL-c	Low-density lipoprotein cholesterol
CCL2	C-C motif chemokine ligand 2
NNS	Non-nutritive sweetener
ACOX	Peroxisomal acyl-coenzyme A oxidase
PPARα	Peroxisome proliferator-activated receptor-α
SREBP1	Sterol regulatory element binding protein-1
TC	Total cholesterol
TG	Triglycerides
TNF-α	Tumor necrosis factor-α
